# Pulmonary Blastoma: A Case Report and Review of the Literature

**DOI:** 10.1002/ccr3.70527

**Published:** 2025-06-25

**Authors:** Haseeb Ahsin, Farwa Aftab, Alishba Amjad, Sumbal Saeed, Fattahullah Khan

**Affiliations:** ^1^ Shaukat Khanum Memorial Cancer Hospital and Research Centre Peshawar Pakistan; ^2^ Khyber Girls Medical College Peshawar Pakistan; ^3^ Khyber Medical University Peshawar Pakistan

**Keywords:** lung cancer, oncology, pulmonary blastoma, pulmonology

## Abstract

This report aimed to underscore the significance of pulmonary blastoma, a rare entity with limited literature, by contributing insights into its clinical features, diagnostic approaches, and treatment modalities. Additionally, it emphasizes the critical role of a multidisciplinary approach in the effective management of this complex and challenging disease.

## 1 Background

Pulmonary blastomas are rare and aggressive lung tumors that make up 0.25%–0.5% of primary lung cancers and have a poor prognosis. They resemble tissue from the fetal lung and have both mesenchymal and epithelial components in their biphasic histology. Recent changes in classification by the World Health Organization (WHO) have separated well‐differentiated fetal adenocarcinoma (WDFA) and malignant pleuropulmonary blastoma (PPB) from classic biphasic pulmonary blastoma (BPB). This reclassification, along with their rarity, complicates the understanding of their epidemiology and clinical characteristics. The text discusses a clinical case and literature review, highlighting treatment challenges and the reliance on limited observational data and case reports for guidance.

## 2 Case Presentation

A 41‐year‐old male presented to the outpatient department with the chief complaints of right‐sided chest pain for 2 months and mild breathlessness on exertion for 1 month. He had no other complaints of fever, cough, weight loss, anorexia, hemoptysis, or paroxysmal nocturnal dyspnea, but with time, his disease progressed, and he developed mild cough, pleural effusion, and hypoxia. He was a former smoker for about 15 pack‐years. He also used to take alcohol occasionally 5 to 6 years back but then stopped completely. He was a known case of HIV diagnosed back in 2019 and was under treatment with ARV and an undetectable viral load since 07/02/2023. He got spontaneous pneumothorax in the left lung at the age of 23 for which he was treated via chest tube insertion. He had an RTA back in 2005. On examination, he looked well, had no palpable lymph nodes, no oral thrush, no clubbing, a clear chest, but absent breath sounds on the right side on auscultation.

### 2.1 Investigations and Treatment

**FIGURE 1:**
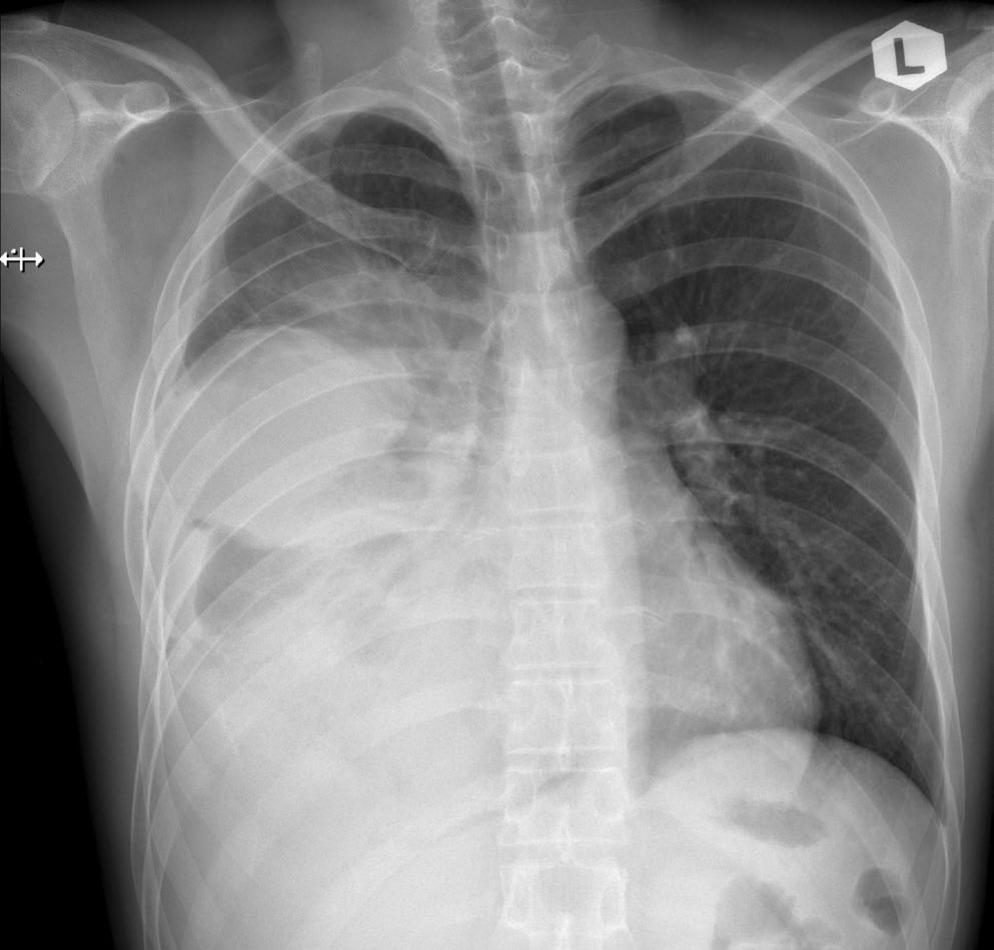
Chest radiograph (posteroanterior view) showing a large mass in the right lung with features suggestive of a pulmonary malignancy.

**FIGURE 2:**
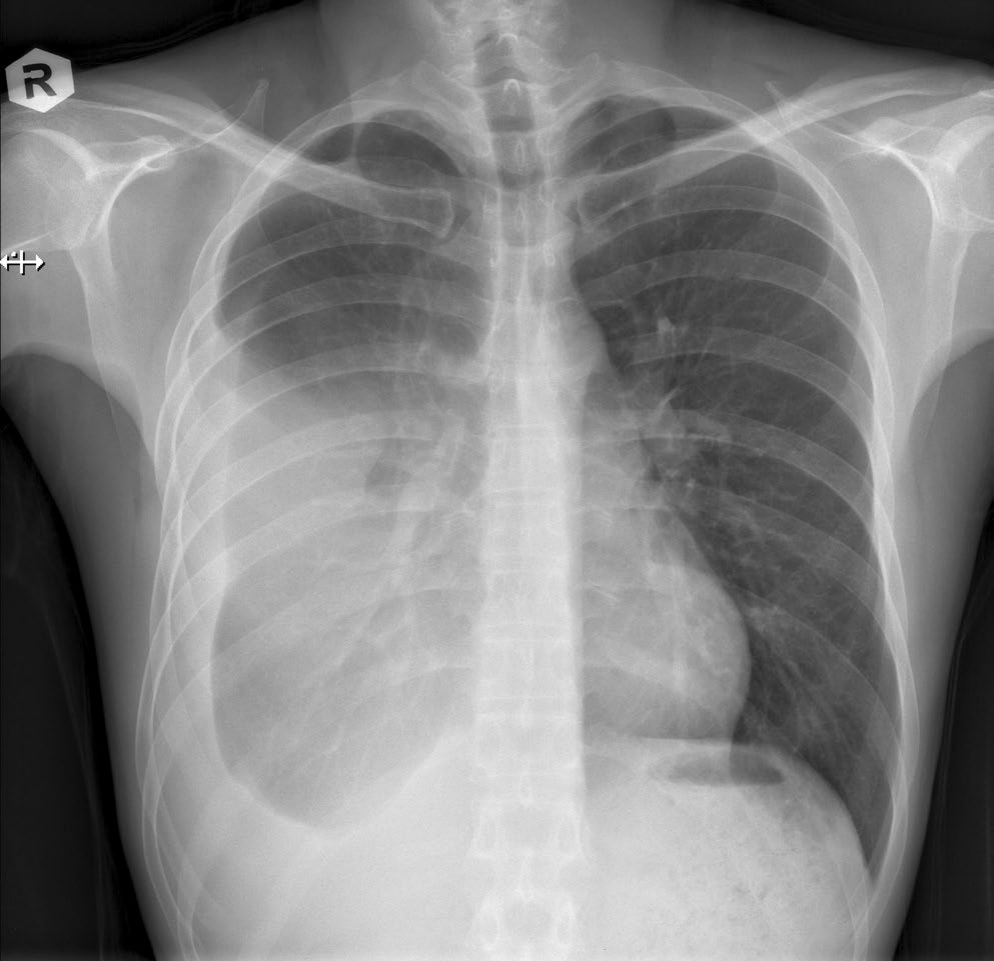
Chest radiograph (posteroanterior view) showing a large mass in the right lung with features suggestive of a pulmonary malignancy.

**FIGURE 3:**
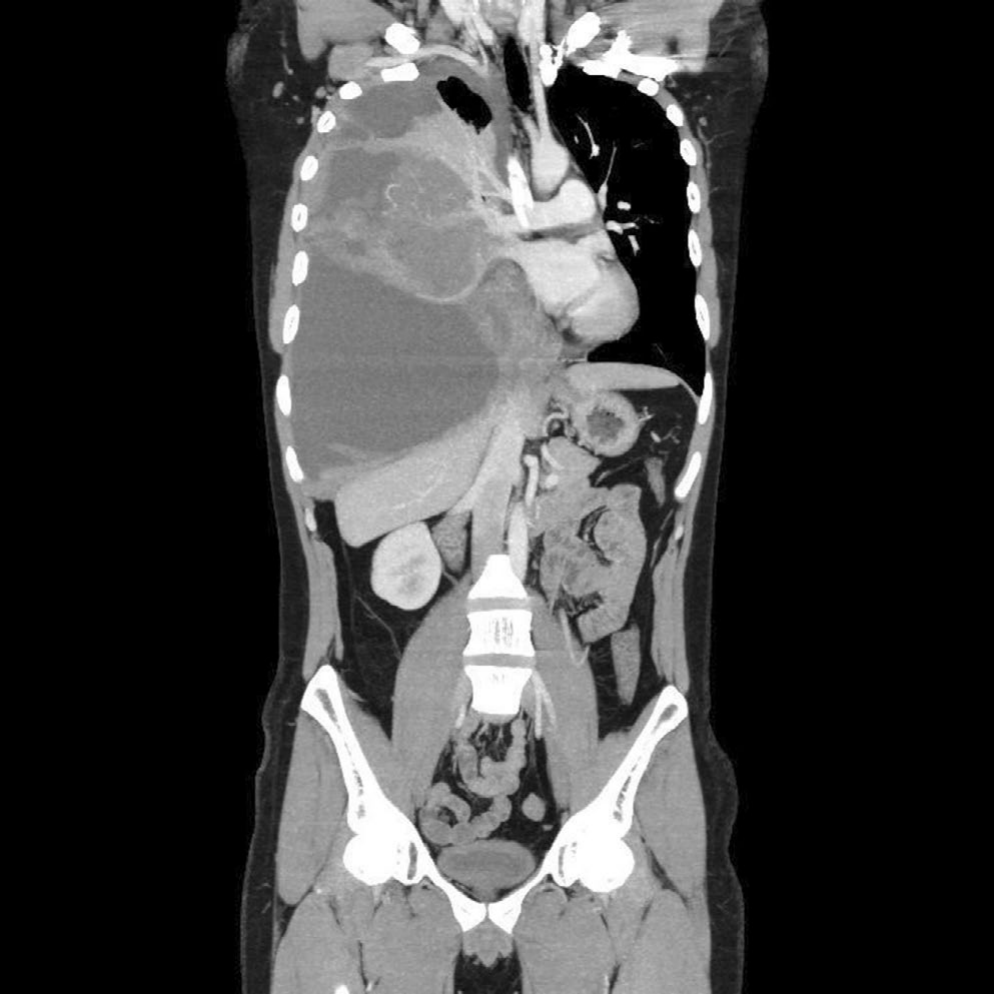
CT scan of the chest (coronal view) showing a heterogeneous right upper lobe pleural‐based mass with mixed solid and cystic components.

**FIGURE 4:**
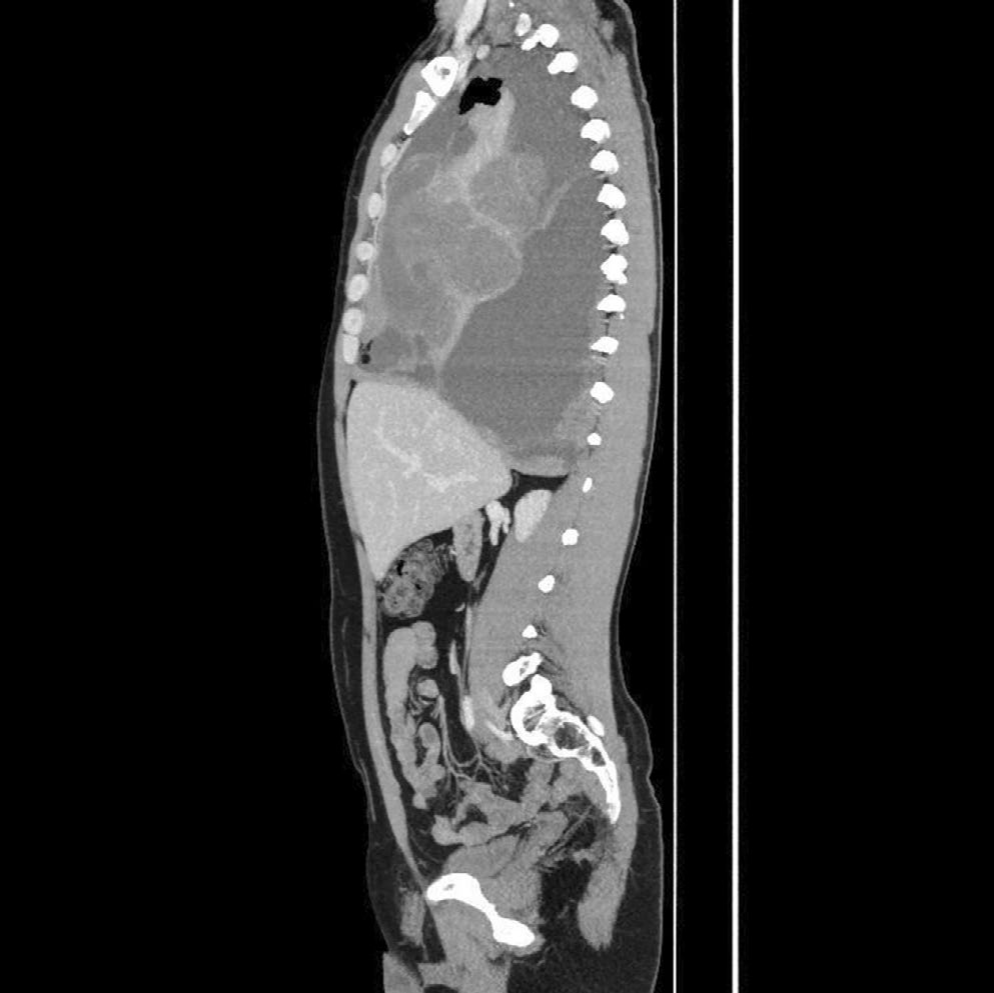
CT scan of the chest (sagittal view) showing a heterogeneous right upper lobe pleural‐based mass with mixed solid and cystic components.

A mass was seen in the right upper and middle lobe on a chest radiograph. (see Figures 1 and 2) A subsequent CT of the chest showed a right upper lobe pleural‐based mass. (see Figures 3 and 4).

**FIGURE 5:**
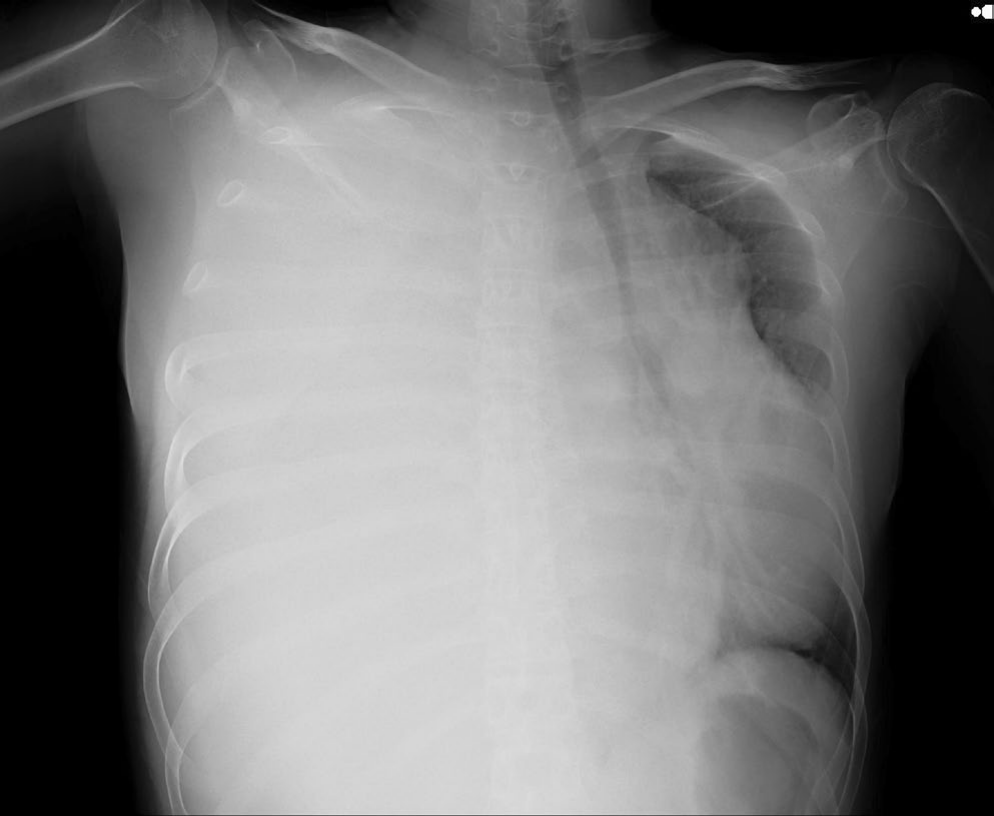
Follow‐up chest radiograph (posteroanterior view) illustrating further progression of the mass in the right lung and involvement of left lung.

A core biopsy using CT guidance of the right lung mass was performed, which gave an impression of adenocarcinoma of the lung initially. A biopsy performed a month later revealed a scanty focus of a malignant neoplasm composed of nests of atypical, hyperchromatic cells. Adenocarcinoma with solid and acinar patterns, as well as adenocarcinoma with acinar patterns and mesenchymal components (round and spindle‐shaped cells) were all made more likely by morphology. Due to the limited nature, prognostic indicators and molecular tests could not be conducted. A repeated biopsy revealed a malignant neoplasm with a biphasic morphology, comprising a hypercellular spindled stroma, desmin negative, SS18‐SSX negative, and B catenin focal positive, features favoring pulmonary blastoma. PET CT scan showed a large heterogeneously enhancing, lobulated right lung mass involving the right upper and middle lobes, with some internal component of necrosis that appeared photopenic. The mass measured 13.4 x 7.6 x 11.9 cm (AP x TR x CC), SUV 12.9. It was invading the chest wall anteriorly at the level adjacent to the right 3rd rib without any obvious local bone erosion. No size significant or hypermetabolic mediastinal lymphadenopathy was appreciated. No suspicious pulmonary nodules in the rest of the lungs. No pleural effusion was seen. Bilateral axillary recesses appeared unremarkable. Sections through the brain parenchyma showed normal physiological tracer uptake without any focal abnormality. No significant and metabolically active bilateral cervical lymphadenopathy was appreciated. A tiny right supraclavicular node was appreciated, which measured 0.5 cm and was nonavid. Liver, spleen, pancreas, bilateral kidneys, and adrenals appeared unremarkable. There was no significant and metabolically active abdominopelvic lymphadenopathy. Physiological FDG uptake was noted in the bowel without any locality. Bilateral inguinal recesses were clear. Homogeneous tracer distribution was noted throughout the visualized skeleton. No destructive osseous lesions. In short, no hypermetabolic locoregional or distant metastasis. A repeated CT chest and abdomen with contrast showed interval increase, measuring 19 x 11 cm in the axial plane and 27 cm in its CC dimension. Previously, it measured 17 x 11 cm and 17 cm, respectively. It displayed several enhancing septations and a mixed solid cystic look. The right hemidiaphragm and liver were being inferiorly displaced by the mass that was spreading across the midline with a mediastinal shift to the left. SVC and IVC were pushed by the mass, but they were patent. The right lung was encased by the mass; its upper lobe was relatively aerated while middle and lower lobes collapsed due to mass effect. The left lung showed normal aeration. No suspicious pulmonary nodules seen. No pneumothorax noted. This interval increase in the size of the mass indicated disease aggressiveness and progression. Involvement of the mediastinum made it T4 disease. Predicted stage was T4 N0 M0. Serial CT scans performed subsequently showed further progression with increasing areas of necrosis, increasing left cardiomediastinal shift, and infiltration into the right pulmonary vein. There was also interval development of multifocal patches of ground glass appearance in the left lung lower lobe. (see Figure 5)] The patient was initially assessed for resection of the mass, and it was found to be unresectable. He was then started on chemotherapy (Carboplatin/Paclitaxel), 12 cycles of which were completed until the patient had stable disease on CT. He was offered surgery but explained that the tumor was borderline resectable.

### 2.2 Conclusion and Results

A month later, the patient developed worsening symptoms and signs of progression on CT, after which XRT (chest wall) was planned. The patient was later put on symptomatic treatment and end‐of‐life care pathway owing to deteriorating clinical condition.

## 3 Discussion

Pulmonary blastoma is a rare and aggressive neoplasm of the lung, accounting for approximately 0.5% of all primary lung tumors [1]. They have morphological similarities to lung tissue from fetuses before 4 months of pregnancy [2]. Despite its rare occurrence, existence of Pulmonary blastoma is well documented [3]. PB was initially characterized by Barnett and Barnard in 1945 under the term “embryonoma.” [4, 5] In 1961, Spencer named the cancer due to the resemblance of its microscopic appearance to that of fetal lung tissue during the 10–16 week stage of development, known as the para‐adenomal stage of lung development [6, 7]. Spencer was the first to refer to the tumor as “pulmonary blastoma,” assuming it was the pulmonary equivalent of nephroblastoma, and he recorded three occurrences [6]. An epithelial type of pulmonary blastoma with malignant yet well‐organized glandular epithelium displaying embryonic traits was more recently reported by Kradin et al. [8] and Kodama et al. [9]. The latter group referred to this tumor as well‐differentiated adenocarcinoma that mimics fetal lung tubules [9]. Originally, pulmonary blastoma was described as a lung tumor that combined elements of sarcoma and carcinoma, with glandular and mesenchymal components that resemble embryos. Pulmonary blastoma was originally defined as a neoplasm of the lung. According to 2015 World Health Organization (WHO) Classification, PB has been classified under pulmonary sarcomatoid carcinoma which is a heterogeneous category of primary lung cancer [10]. A variety of malignant epithelial tumors (carcinomas) that histologically resemble sarcomas and either have or do not have the typical characteristics of non‐small cell lung cancer (NSCLC) are referred to as pulmonary sarcomatoid carcinoma (PSC) [10]. Therefore, this rare category of pulmonary neoplasms encompasses pleomorphic carcinoma, spindle cell carcinoma, giant cell carcinoma, pulmonary blastoma, and carcinosarcoma [10]. Childhood lung tumors are subdivided into three subtypes pulmonary blastoma, fetal adenocarcinoma, and pleuropulmonary blastoma (PPB) [11]. The presence of both malignant and immature epithelial components is a characteristic of fetal adenocarcinomas [11]. In contrast, pleuropulmonary blastoma is defined by a malignant and immature mesenchymal proliferation [11]. The blastoma exhibits biphasic features, comprising both mesenchymal and epithelial malignant immature components that resemble lung tissue of 10 to 16 weeks of gestation [11].

### 3.1 Clinical Presentation

Patient usually presents with complaints of cough, hemoptysis, dyspnea, chest pain occurring because of the mass compressing the bronchi or pleura, fever, weight loss, and recurrent pneumonia [3, 12]. Occasionally, spontaneous pneumothorax and neurological symptoms can develop too [12]. The tumors are frequently discovered by chance on chest radiographs, and about 40% of people with this illness have no symptoms [3, 12]. Clinical evaluations may show reduced breath sounds in specific areas or other effects associated with cigarette smoking, with more than 80% of cases linked to a smoking history or asbestos exposure. The review by Van Loo indicates that the average age of CBPB patients is 39 years, and the male‐to‐female ratio is 1.5:1 [12].

### 3.2 Diagnosis

Laboratory investigation abnormalities have been infrequent and nonspecific, and there is no existing literature detailing specific laboratory findings associated with pulmonary blastoma. Pulmonary blastoma commonly appears as a large, well‐defined unilateral mass in the peripheral lung, as noted in studies by Lee [13] and Koss et al. [3] Due to its location, a tissue diagnosis via bronchoscopy is only possible in about 25% of cases [3]. However, these tumors can be effectively visualized on thoracic ultrasound, with imaging results that align with CT findings [14]. Typical imaging characteristics include round or oval solid masses with smooth edges and lobed borders, usually without “burr” formation [15]. Tumor invasion into adjacent structures can lead to complications such as obstructive pneumonia, pleural effusion, or atelectasis, complicating diagnosis and potentially leading to misdiagnosis in clinical practice [15]. Pulmonary blastoma on CT scans is identified by its dense and vesicular structure, which shows varying contrast uptake and features central necrosis [12]. It is unclear how PET‐CT is used to stage pulmonary blastoma, although some cases support their findings in terms of pathological staging. Due to the complex histology, preoperative diagnoses succeed in only about one‐third of cases [1]. It is critical to differentiate pulmonary blastoma from malignant tumors, such as primary and metastatic lung malignancies, as well as benign diseases like pleural fibroma and hamartoma [16]. Immunohistochemical staining is crucial for diagnosing pulmonary blastoma (PB). Tumor cells in the epithelial component test positive for keratin, epithelial membrane antigen, and TTF‐1. Neuroendocrine cells within the tumor express CD56, Syn, or CgA. β‐catenin shows nucleus‐positive staining in glands and morula bodies, while vimentin is positive in mesenchymal components, with additional specific markers potentially found in heterodifferentiated areas [15]. In the case presented above, biopsy showed beta‐catenin focal positivity as well owing to pulmonary blastoma diagnosis.

### 3.3 Histology

Microscopically, the epithelial components of pulmonary blastoma (PB) resemble those of fetal lung tissue from 11 to 18 weeks of gestation, characterized by branched or back‐to‐back glandular hyperplasia, columnar cells with reduced cytoplasm, and small, uniform, round to oval nuclei with minimal atypia. Morula bodies are present in approximately 40% of PB cases [15]. The mesenchymal differentiation features include primitive oval or spindle cells, with allogenic differentiation, such as skeletal muscle, bone, and cartilage, observed in about 25% of cases. Occasional nuclear polymorphism and nuclear fissions may be noted. Additionally, adenocarcinoma or poorly differentiated carcinoma areas can be found in 30% to 50% of PB patients [15, 17].

### 3.4 Management

Currently, there is no standard management for pulmonary blastoma (PB). Patients may benefit from a combination of surgery, radiotherapy, and chemotherapy. Surgical resection is typically the primary treatment for localized cases. Similar to non‐small cell lung cancer (NSCLC), lobectomy is the most commonly performed surgical procedure for pulmonary blastoma patients [18, 19, 20]. For patients with late‐stage disease, a comprehensive treatment strategy may be considered, incorporating surgery, chemotherapy, and radiotherapy [21]. While no single agent has been proven to be more effective than others, cisplatin is commonly utilized due to its effectiveness against primitive tumors [16]. The effectiveness of adjuvant therapy has not been confirmed through clinical trials; however, several case reports indicate positive outcomes with the use of adjuvant chemotherapy, either alone or in combination with radiotherapy [22]. A literature review has suggested that a regimen of cisplatin and etoposide may be suitable for adjuvant chemotherapy [23]. In a more recent study by Lewis et al. (2018), two patients who underwent surgical treatment received four cycles of adjuvant cisplatin‐based chemotherapy followed by thoracic radiation, and both patients experienced long‐term survival. [24] Metastasis occurs in about 43% of cases, frequently affecting the brain, mediastinum, pleura, diaphragm, liver, soft tissues in the limbs, submandibular glands, scrotum, and ovaries [23, 24]. In the context of metastatic disease, chemotherapy is the primary treatment option; however, there are no established guidelines for specific regimens. Reviews by Cutler et al. (1998) [24] and more recently by Lewis et al. (2018) [25] have examined cases of patients who underwent chemotherapy. Initially, single‐agent chemotherapy was attempted but yielded no clinical or objective responses [23]. Vila et al. were pioneers in using combination chemotherapy with chlorambucil and methotrexate in 1973 [25]. Over the years, various cytotoxic regimens have been employed, including cisplatin‐etoposide, with or without ifosfamide, as well as cyclophosphamide and vincristine‐based treatments [22]. Other commonly used chemotherapeutic agents include carboplatin, doxorubicin, and paclitaxel. [22] There are only a limited number of reports on the molecular alterations found in PB, and even fewer that are suitable for targeted therapies [22]. Two cases have been documented where the tumor exhibited a ROS1 rearrangement. In the first case, fluorescence in situ hybridization (FISH) revealed a ROS1 rearrangement in 7 out of 50 tumor cells (14%) [26]. In the second case, the patient had a detectable CD74–ROS1 rearrangement and showed a positive response to crizotinib, presenting a new treatment option for PB [27]. Evidence regarding other molecular alterations is still limited; however, in the absence of established therapies and considering the adenocarcinoma component of the tumor, it is reasonable to investigate potential targetable mutations [28]. Additionally, while some cases of PB have shown high expression of PD‐L1, there have been no published studies on the use of immunotherapeutic agents for this condition [29].

### 3.5 Prognosis

The prognosis for pulmonary blastoma is generally poor, with two‐thirds of patients dying within 2 years and a five‐year survival rate of only 16% [16]. Tumor size at diagnosis is a key factor influencing prognosis, with tumors smaller than 5 cm associated with better outcomes [16]. Poor prognosis is also linked to tumor metastasis and recurrence despite surgical resection [16]. Notably, 43% of tumors recur within the first year, often spreading to locations such as the brain and mediastinum [3]. Recurrence typically occurs within the first year following diagnosis or not at all [30].

## 4 Conclusion

We present a case of classic biphasic pulmonary blastoma, a rare type of lung cancer that typically affects younger patients and is linked to a poorer outcome compared to more common lung cancers. Pulmonary blastoma is an infrequent and aggressive cancer characterized by a poor outlook. Its rapid growth and biphasic nature complicate diagnosis, and any delays in identifying the condition can adversely impact patient outcomes. The absence of a specific treatment for this cancer poses a significant challenge, as selecting the right therapeutic agents is complicated by its intricate structure, which requires a combination of therapies targeting different components. Moreover, the rarity of this cancer complicates the establishment of an effective treatment plan. As a result, treatment regimens must be tailored to the individual needs of each patient. Pulmonary blastoma (PB) displays distinctive microscopic characteristics reminiscent of fetal lung tissue at 11 to 18 weeks of gestation. Key features include branched glandular hyperplasia and columnar cells with small uniform, round to oval nuclei with minimal atypia. Given the rarity of these cases, there is a critical need for multicenter collaboration to create databases, facilitate large clinical trials, and develop therapeutic guidelines.

## Author Contributions

**Haseeb Ahsin:** conceptualization, formal analysis, project administration, supervision, validation, writing – review and editing. **Farwa Aftab:** data curation, investigation, writing – original draft. **Alishba Amjad:** investigation, writing – original draft. **Sumbal Saeed:** investigation, writing – original draft. **Fattahullah Khan:** supervision, writing – review and editing. All authors have read and approved the final version of the manuscript and agree to be accountable for all aspects of the work, ensuring accuracy and integrity.

## Ethics Statement

Written informed consent was obtained from the patient's brother, as the patient was deceased, to publish this case report in accordance with the journal's patient consent policy. The study utilized anonymized data from the Hospital Information System.

## Conflicts of Interest

The authors declare no conflicts of interest.

## Data Availability

The data that support the case report was obtained from the Hospital Information System of Shaukat Khanum Memorial Cancer Hospital and Research Center Peshawar, after obtaining the required permissions. Restrictions apply to the availability of this data. Data are available from the corresponding author with the permission of Shaukat Khanum Memorial Cancer Hospital and Research Center Peshawar.
